# Sensory Neuron-Derived Eph Regulates Glomerular Arbors and Modulatory Function of a Central Serotonergic Neuron

**DOI:** 10.1371/journal.pgen.1003452

**Published:** 2013-04-18

**Authors:** Ajeet Pratap Singh, Rudra Nayan Das, Gururaj Rao, Aman Aggarwal, Soeren Diegelmann, Jan Felix Evers, Hrishikesh Karandikar, Matthias Landgraf, Veronica Rodrigues, K. VijayRaghavan

**Affiliations:** 1 National Centre for Biological Sciences, Tata Institute of Fundamental Research, GKVK Campus, Bangalore, India; 2 Department of Zoology, University of Cambridge, Cambridge, United Kingdom; 3 Department of Biological Sciences, Tata Institute of Fundamental Research, Mumbai, India; New York University, United States of America

## Abstract

Olfactory sensory neurons connect to the antennal lobe of the fly to create the primary units for processing odor cues, the glomeruli. Unique amongst antennal-lobe neurons is an identified wide-field serotonergic neuron, the contralaterally-projecting, serotonin-immunoreactive deutocerebral neuron (CSDn). The CSDn spreads its termini all over the contralateral antennal lobe, suggesting a diffuse neuromodulatory role. A closer examination, however, reveals a restricted pattern of the CSDn arborization in some glomeruli. We show that sensory neuron-derived Eph interacts with Ephrin in the CSDn, to regulate these arborizations. Behavioural analysis of animals with altered Eph-ephrin signaling and with consequent arborization defects suggests that neuromodulation requires local glomerular-specific patterning of the CSDn termini. Our results show the importance of developmental regulation of terminal arborization of even the diffuse modulatory neurons to allow them to route sensory-inputs according to the behavioural contexts.

## Author Summary

Serotonin, a major neuromodulatory transmitter, regulates diverse behaviours. Serotonergic dysfunction is implicated in various neuropsychological disorders, such as anxiety and depression, as well as in neurodegenerative disorders. In the central nervous systems, across taxa, serotonergic neurons are often small in number but connect to and act upon multiple brain circuits through their wide-field arborization pattern. We set out to decipher mechanisms by which wide-field serotonergic neurons differentially innervate their target-field to modulate behavior in a context-dependent manner. We took advantage of the sophisticated antennal lobe circuitry, the primary olfactory centre in the adult fruitfly *Drosophila melanogaster*. Olfactory sensory neurons and projection neurons connect in a partner-specific manner to create glomerular units in the antennal lobe for processing the sense of smell. Our analysis at a single-cell resolution reveals that a wide-field serotonergic neuron connects to all the glomeruli in the antennal lobe but exhibits the glomerular-specific differences in its innervation pattern. Our key finding is that Eph from sensory neurons regulates the glomerular-specific innervation pattern of the central serotonergic neuron, which in turn is essential for modulation of odor-guided behaviours in an odor-specific manner.

## Introduction

Serotonin, 5-hydroxytryptamine (5-HT), an evolutionarily ancient monoamine, plays diverse roles in the brain [1], [2], [3]. In the mammalian brain, serotonin is implicated in the regulation of behavioural arousal and control of motor output [4], [5] with a proposed phylogenetically ancient function in modulating a drive to withdraw from dangerous and aversive environments and seek contentment [6]. In the fruitfly, *Drosophila melanogaster*, serotonin regulates diverse aspects of behaviour such as aggression, sleep, circadian rhythm, learning and memory [7], [8], [9], [10], [11]. It is estimated that there is one serotonergic neuron per million in the mammalian central nervous system, yet, when axon terminals are examined in the rat cortex, as many as 1/500 are serotonergic [2], suggesting that a small set of neurons may act through their broad arborization pattern to play roles in modulating many brain circuits. Understanding how serotonin and other neuromodulators function to modify intrinsic dynamic properties of neuronal circuits and thereby alter animal behaviour, is a daunting task. An iconic preparation in which this has been carried out is the circuit that drives pyloric rhythm in the crab/lobster stomatogastric system [12], [13]. Such studies have led to the view that understanding the function of brain circuits not only requires a characterization of intrinsic dynamic properties of constituent neurons and their connectivity but also an understanding of how specific neurotransmitters and neuromodulators impinge on the circuit [14].

Functional imaging and electrophysiology suggests that serotonergic modulation of olfactory information is an important conserved feature [15], [16], [17]. In the *Drosophila* antennal lobe (AL), innervated by ∼2500 olfactory sensory neurons (OSNs), ∼150 projection neurons (PNs), and ∼200 local interneurons (LNs), the CSDn is the sole serotonergic neuron [18], [19], [20]. This and its accessibility to genetic manipulation [18], [21] allow the development of the capacity for serotonergic modulation to be studied in the context of the well-characterized olfactory glomerular system.

While the CSDn's axonal terminals spread over multiple glomeruli in the adult AL [18], it also exhibits glomerular-specific differences in innervation pattern (this study). Such wide-field arborizations, with variations in specific glomeruli, are seen in multi-glomerular olfactory LNs [22], [23], but the underlying mechanisms that regulate these arborizations have not been studied. This is in contrast with the many elegant studies that have led to significant understanding of mechanisms underlying targeting of the uni-glomerular OSNs and PNs [24], [25], [26]. The glomerular-specific pattern of wide-field interneurons is also likely to be important for their function as context- specific modulators of olfactory information, a hypothesis that has not been tested. Serotonergic neurons have been suggested to act in a paracrine manner: serotonin-containing varicosities release serotonin that can diffuse away and act on extra-synaptically located receptors [27]. While the arbors of such diffuse neuromodulatory neurons are suggested to be distributed to optimize efficient coverage of brain regions, the heterogeneous distribution of the terminal arbors of the CSDn in the AL suggests the possibility that arborization in a specific glomeruli is an important functional feature and could be behaviourally relevant, a view which we test and show to be valid.

In searching for the mechanistic underpinning of the CSDn's terminal aroborization pattern we homed in on Eph-ephrin signaling as a likely candidate. Eph receptors (Eph) form the largest family of receptor tyrosine kinases (RTKs) and mediate contact-dependent bidirectional communication between cells through short-range interactions [28], . Such short-range interactions between axonal arbors and their target cells could be relevant for emergence of regional differences in the arborization pattern of neurons in the CNS. We find that an Eph/ephrin signaling-mediated repulsion plays a key role in glomerular-specific positioning of axonal terminals of the CSDn. Sensory neurons differentially express Eph, which interacts with Ephrin on the CSDn to establish glomerular-specific innervation pattern of the CSDn axonal terminals. Further, we show that this glomerular-specific innervation pattern of the CSDn allows it to modulate olfactory behaviour in an odor-specific manner.

We have determined the function of the CSDn in modulating odor-guided behaviour and shown that its glomerular-specific modulatory properties are dependent on the developmental regulation of its terminal arborization. Since the CSDn is the only serotonergic neuron in the AL, our study behaviourally dissects out the role of this important neuromodulator in the olfactory system and shows, for the first time, how its function is developmentally put in place. Our results also point to how sensory neurons, which are targeted to specific glomeruli, could locally regulate terminal arbors of other wide- field neurons. Finally, we examine Eph-ephrin signaling at the resolution of a single neuron, for the first time, to show how short-range signaling can sculpt local pattern, and thereby, function.

## Results

### The glomerular-specific arborization of a central serotonergic neuron autonomously requires Ephrin

**Figure 1:**
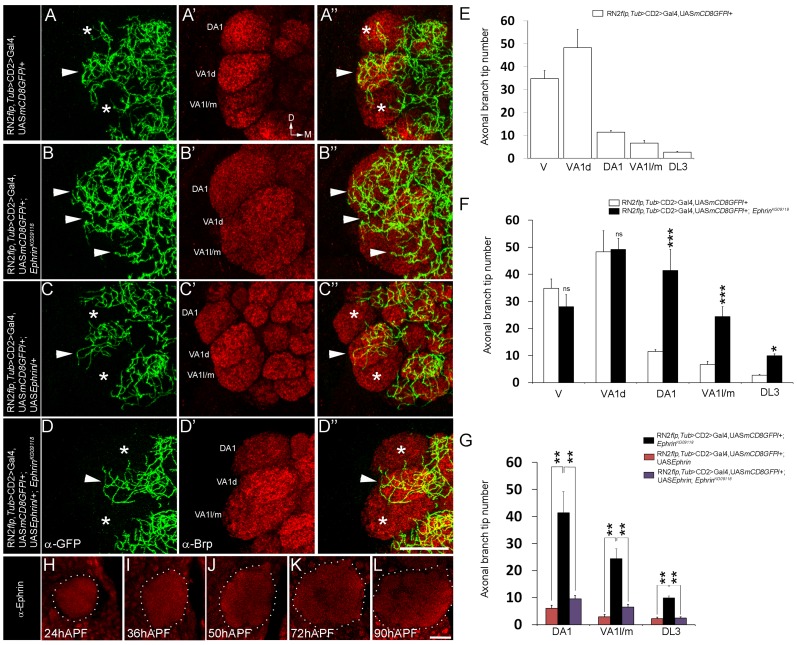
Glomerular-specific innervation pattern of the CSDn in the AL is regulated by Ephrin. (A-A″, E) Innervation pattern of the axonal terminals of the CSDn (green) in glomeruli VA1l/m, VA1d and DA1 (anti-Brp in red) in control adults is shown (n>6). Asterisks indicate glomeruli with fewer innervations and arrowhead indicates glomerulus with more innervations from the CSDn. (B-B″, F) In *Ephrin^KG09118^* hypomorphs, increased terminal innervations can be seen to VA1l/m (n = 5, p<0.001), DA1 (n = 5, p<0.001) and DL3 (n = 9, p = 0.018) while innervations in VA1d (n = 5, p = 0.865) and V (n = 4, p = 0.149) are comparable to controls. (D-D″, G) Targeted expression of Ephrin in the CSDn in *Ephrin^KG09118^* hypomorphs restores distribution of axonal terminals in VA1l/m (n = 6, p = 0.99), glomerulus DA1 (n = 6, p = 0.606) and glomerulus DL3 (n = 6, p = 0.992). (C-C″, G) Targeted expression of Ephrin in the CSDn does not change overall distribution pattern of axonal tips in VA1l/m (n = 8, p = 0.241), DA1 (n = 8, p = 0.092233) and DL3 (n = 8, p = 0.910) when compared to controls, however a small decrease in overall branch tip number is observed. (E–G) Quantification of total axonal branch tip number in glomeruli V, VA1l/m, VA1d, DA1 and DL3 is plotted in histograms. A one-way repeated measure ANOVA test was performed to assess significant difference between the genotypes (F = 28.544, P<0.001). All pairwise multiple comparisions were performed using Fisher LSD method.. *, p<0.05; **, p<0.01; ***, p<0.0001; n.s. (not significant), p>0.05. (H–L) Ephrin shows broad expression pattern and it is expressed throughout the developing AL (n>5). APF = After puparium formation. All the images hereafter are oriented as indicated in A′ unless otherwise mentioned. D, dorsal; M, medial. Scale bar = 20 µm. See also Table S1.

We had earlier characterized the development the CSDn in *Drosophila* [18], [21]. In these studies, the CSDn [18] is labeled using a combination of *cis*-FRT/FLP and Gal4/UAS method [31], [32]. This method can result in activation of CD8::GFP reporter protein expression in the CSDn in one antennal lobe, while the neuron on the contralateral side remains unlabeled, thereby allowing the examination of its arbors without the pattern being obscured by its homolog in the other hemisegment. Although the CSDn's terminal arbors in the contralateral AL innervate all glomeruli [18], a closer examination showed clear glomerular-specific differences in the innervation pattern (Figure 1A, 1E). We focused on glomeruli whose function in olfactory perception is well established in behavioural assays allowing us to correlate connectivity of the CSDn with its function in modulating behaviour. We therefore analyzed the VA1d, DA1, VA1l/m, DL3, which respond to fly- derived odors [33]. Of these, sensory neurons innervating DA1 and DL3 respond to the pheromone *cis*-vaccenyl acetate - cVA [33], [34], [35]. We also examined the V glomerulus, which responds to Carbon dioxide (CO_2_) [36], [37]. Quantification of axonal branch tip number of the CSDn in these glomeruli demonstrated prominent glomerular-specific differences in its innervation pattern: VA1d and V were innervated by many arbors while DA1, VA1l/m and DL3 received fewer inputs from the CSDn (Figure 1A, 1E; Figure S1 and Table S1). In order to understand the cellular and molecular mechanism(s) underlying such differences in innervation pattern of the wide-field neuron we analyzed the possible role of signaling molecules and observed a clear disruption of this pattern in *Ephrin* hypomorphs (Figure 1B, 1F; Figure S1 and Table S1). Axonal branch tip number increased dramatically in DA1, VA1l/m and DL3 glomeruli of *Ephrin* hypomorphs while innervations to glomeruli VA1d and V is comparable to controls (Figure 1B, 1F; Figure S1 and Table S1): The glomeruli that normally had fewer arbors of the CSDn (DA1, VA1l/m and DL3) were densely innervated in *Ephrin* hypomorphs, whereas arbors in densely innervated glomeruli (VA1d and V) remained unchanged in this mutant. Further, CSDn-specific expression of Ephrin rescued glomerular-specific innervation pattern defects observed in *Ephrin* hypomorphs (Figure 1C, 1D, 1G; Figure S1 and Table S1) suggesting that Ephrin is required autonomously in the CSDn although it is widely expressed in the developing AL (Figure 1H–1L). Overexpression of Ephrin in the CSDn did not change overall pattern of axonal branch tip distribution although a small decrease in final branch tip number was observed (Figure 1C, 1G; Figure S1 and Table S1). This reduction in the overall branch tip number could either be due to increased Eph-mediated repulsion or due to other as yet unknown molecular interactions within the AL.

### Eph, the receptor for Ephrin, is differentially expressed by sensory neurons and is capable of initiating repulsive interactions with Ephrin in the antennal lobe

**Figure 2:**
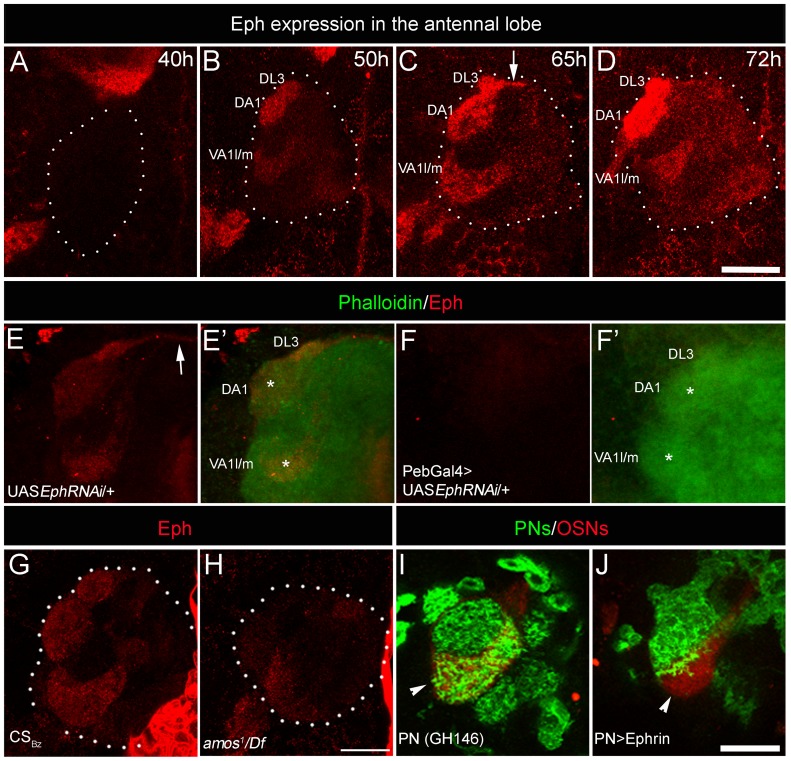
Sensory neurons differentially express Eph, which is capable of initiating repulsive interaction with Ephrin. (A–D) Eph is strongly enriched in three anteriorly positioned glomeruli: VA1l/m, DA1 and DL3 glomeruli in the developing AL starting from 50 hAPF (n>5). White dots encircle the developing AL. Arrow indicates antennal commissure. (F-F′) Targeted expression of *EphRNAi* in sensory neurons (*Pebbled*-Gal4/+; UAS *EphRNAi*/+) results in strong reduction of Eph expression (red) in the antennal lobe compared to (E-E′) controls (UAS *EphRNAi*/+). AL is counterstained with phalloidin (green). (G–H) Eph expression is reduced in the AL of animals lacking majority of the OSNs from trichoid and basiconic sensilla. (G) Eph (red) is prominently expressed in select few glomeruli in the AL at 70 hAPF of control animals. (H) *amos^1^/Df(2L)M36F-S6* animals show drastic reduction in the Eph expression in the AL. (I–J) Targeted expression of Ephrin in PNs prevents their entry in high Eph-expressing glomerulus VA1l/m (arrowhead). (I) In control animals (Gal4-*GH146*,mCD8::GFP/+; Or47b::rCD2/+), PN arbors (green) innervate glomerulus VA1l/m (red). (J) Very few PN arbors innervate VA1l/m glomerulus (red) when Ephrin is overexpressed in PNs (Gal4-*GH146*,mCD8::GFP/UAS *Ephrin*; Or47b::rCD2/+). Scale bar = 20 µm.

While Ephrin was required in the CSDn for positioning its terminal arbors in a glomerular-specific manner (Figure 1A–1D and 1F–1G), expression analysis showed that it is uniformly distributed in the developing AL (Figure 1H–1L) and thus may not provide the positional information for glomerular-specific branching. We therefore examined the expression of Eph, the receptor for Ephrin, in the developing AL. Interestingly, Eph expression, as revealed by an Ephrin-Fc probe [38], was detected in a small subset of glomeruli within the developing AL from 50 h after puparium formation (50 hAPF; Figure 2A–2D). Most prominent Eph expression was detected in DA1, VA1l/m and DL3 glomeruli. These are the same glomeruli that receive fewer arbors of the CSDn in control animals and show substantial increase in innervation by the CSDn in *Ephrin* hypomorphs. The observation of commissural expression of Eph (arrow in Figure 2C and 2E) along with the above glomerular specific pattern suggests that the OSNs are the source of Eph. Consistent with this interpretation, targeted expression of *EphRNAi* in sensory neurons (*pebbled*-Gal4/+; UAS *EphRNAi*/+) abolished Eph expression in the AL (Figure 2E–2F; Figure S2). Targeted misexpression of Eph in sensory neurons (*pebbled*-Gal4/+; UAS *Eph*/+) lead to Ephrin-Fc labeling in the whole AL, further validating the specificity of the Ephrin-Fc probe (Figure S2). Targeted expression of the *EphRNAi* in the projection neurons or in the local interneurons did not affect glomerular-specific Eph expression (data not shown). Furthermore, in *amos* mutant animals, of the genotype *amos^1^/Df(2L)M36F-S6* [39], which lack most OSNs , the AL expression of Eph is also substantially reduced (Figure 2G–2H). Taken together, we conclude that Eph is expressed by a small set of sensory neurons and enriched in cognate glomeruli that received reduced arbors of the CSDn compared to other glomeruli where Eph levels are low.

Ephrin expressed by the CSDn may initiate repulsive interactions upon encountering high levels of Eph on sensory neurons. This hypothesis predicts that high levels of Ephrin ectopically expressed in other interneurons in these glomeruli would result in their arbors being repelled by high Eph expression. To test this hypothesis, we overexpressed Ephrin in PNs and focused our analysis on their arbors in the high Eph-expressing VA1l/m glomerulus, visualized using the *Or47b::rCD2* strain (Gal4-*GH146*,UAS*mCD8::GFP*; *Or47b*::*rCD2*; UAS*Ephrin*). Indeed, targeted overexpression of Ephrin in PNs resulted in a drastic reduction of PN innervations in the VA1l/m glomerulus (Figure 2I–2J), consistent with the view that Eph-ephrin signaling mediates a repulsive interaction within the developing AL. Similar effect of Ephrin misexpression on PN arborization was observed in other high-Eph expressing glomeruli, DL3 and DA1 (Figure S3). This suggests that under normal circumstances, CSDn-derived Ephrin could interact with sensory neuron-derived Eph to appropriately position terminals of the CSDn in a glomerular-specific manner.

### Olfactory sensory neuron-derived Eph regulates glomerular patterning of the CSDn

**Figure 3:**
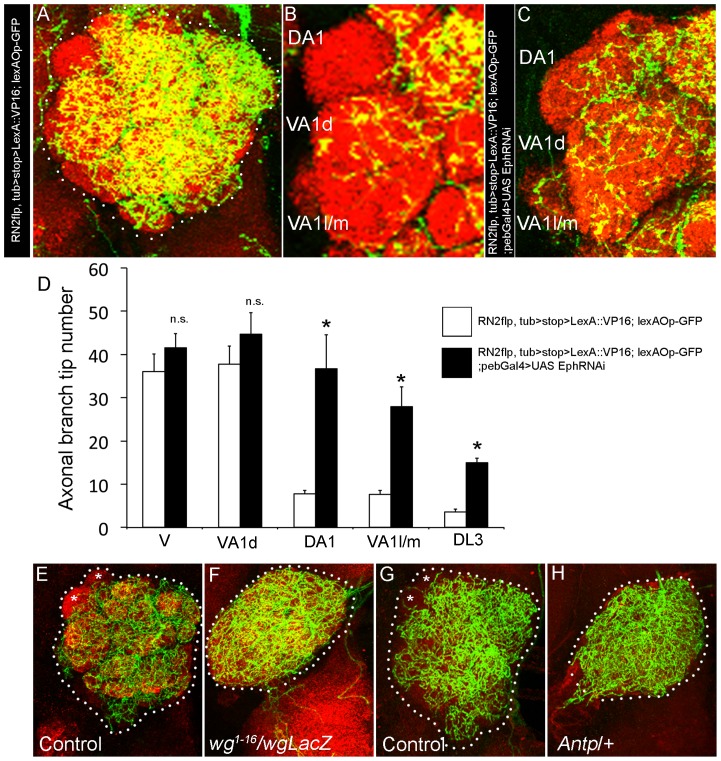
Olfactory sensory neuron-derived Eph controls glomerular-specific arborization pattern of the CSDn. (A) The CSDn is labeled in control (RN2*flp*, *tub*>STOP>LexA::VP16, *lexAOpCD2GFP*) animals (B) which shows distinct glomerular-specific arborization pattern. (C) RNAi-mediated knockdown of Eph in sensory neurons (RN2*flp*, *tub*>STOP>LexA::VP16, *lexAOpCD2GFP*; *Pebbled*-Gal4>UAS *EphRNAi*) leads to increased CSDn arborization in glomerulus DA1 and VA1l/m. (D) Histogram shows quantification of the axonal branch tip number of the CSDn in different glomeruli (n = 3). (E–H) Loss or transformation of antenna leads to uniform arborization of the CSDn in the AL. (E and G) In control animals, terminal arbor of the CSDn shows glomeruli-specific differences in innervation pattern with some glomeruli receiving fewer inputs (asterisks in E and G). Trans-allelic combination of *wg^1-16^* and *wg^LacZ^* leads to loss of antenna and (F) Axonal terminals of the CSDn from animals lacking antenna (*wg^1-16^*/*wg^LacZ^*; RN2*flp*, *tub*>CD2>Gal4, UAS*mCD8GFP*/+) uniformly innervate the AL. (H) In animals where antenna is transformed into leg (RN2*flp*, *tub*>CD2>Gal4, UAS*mCD8GFP*/Antp), axonal terminals of the CSDn innervate the AL homogeneously. Scale bar = 20 µm.

In order to directly assess the role of sensory neuron-derived Eph, we used a combination of Gal4/UAS and LexA/lexAOp dual expression system. We generated RN2*flp*, *tub*>stop>LexA::VP16; *lexAOpCD2*GFP line which labels the CSDn (Figure 3A) and showed a clear glomerular-specific arborization pattern similar to that seen in the GAL4 reporter (Figure 3B, 3D). OSN-specific knockdown of Eph, achieved by targeted expression of *EphRNAi* in OSNs driven by the *pebbled*-Gal4, leads to increased innervation of CSDn in DA1, VA1l/m glomerulus (Figure 3C, 3D; Figure S1) similar to the phenotype that we observed in *Ephrin* hypomorphs (Figure 1). Such a change was also seen for DL3 glomerulus (Figure 3D, Figure S1). These results implicate OSNs in a previously unknown role in the development of a central neuron through their regulated expression of Eph. OSN terminals enter the lobe at 22 h APF and are key components of glomerular development [40]. OSN expression of Eph in the developing antennal lobe becomes prominent after 50 hAPF (Figure 2A–2D). To further validate the role of OSNs in CSDn patterning, we examined the CSDn arborization pattern in animals developing without antennae [41] and thus without the antennal OSNs (Figure 3F) or in animals in which antennae are transformed to legs (Figure 3H). In both the cases, the innervation pattern of CSDn in the antennal lobe was uniform (Figure 3F, 3H), unlike control animals where axonal terminals exhibited glomerular-specific differences in the innervation pattern (Figure 3E, 3G). Taken together, these data substantiate a role for OSNs in providing positional cues necessary for glomerular-specific arborization patterning of an identified central serotonergic neuron.

### Levels of Eph/ephrin signaling regulate positioning of glomerular-specific arbors of the CSDn

**Figure 4:**
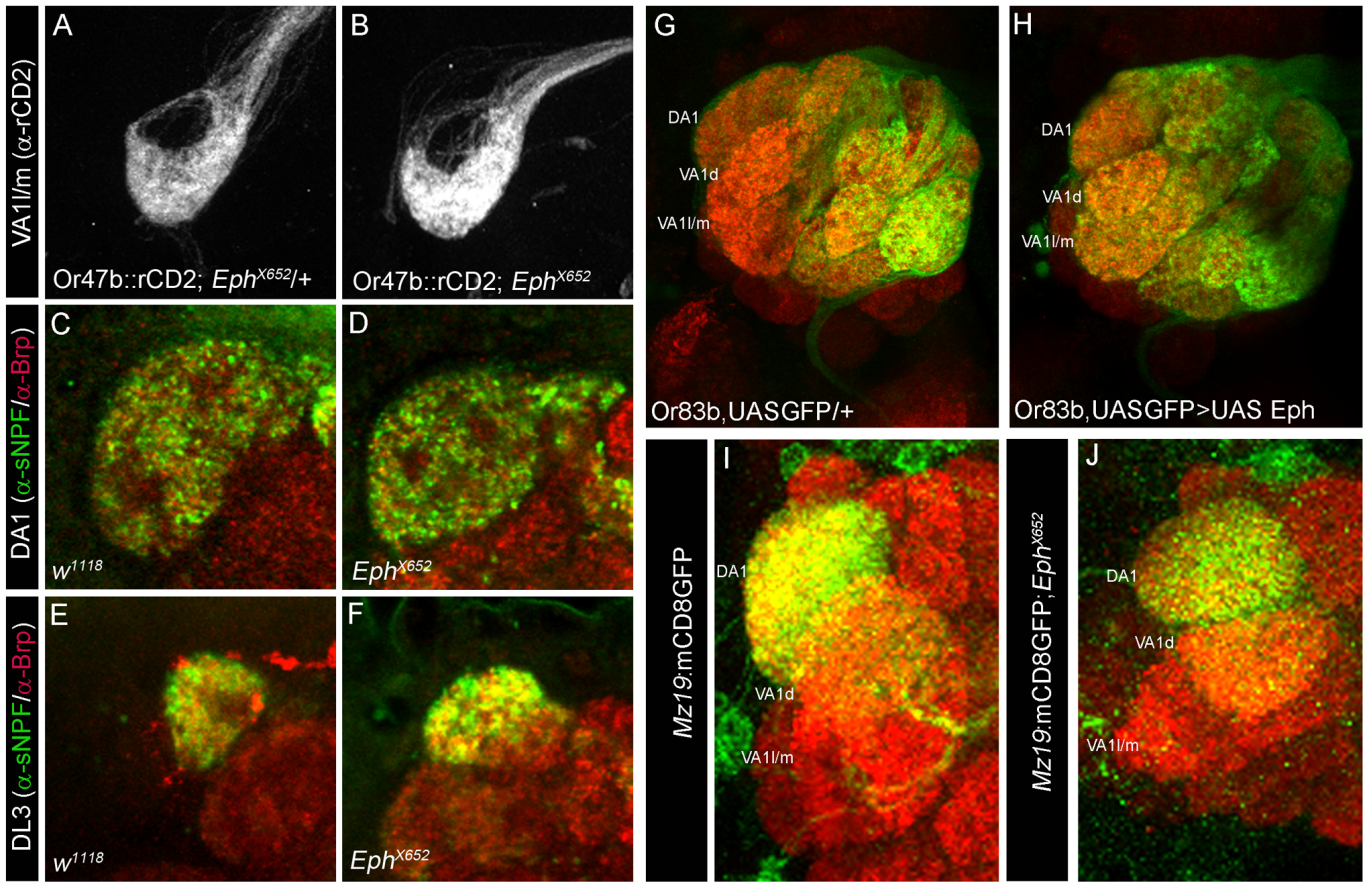
Eph function is not required for appropriate targeting of OSNs and uniglomerular PNs. (A–B) OSN terminals innervating glomerulus VA1l/m appear comparable to (A) controls in (B) *Eph* null animals. (C–F) α-sNPF (green) labels specific sets of OSN terminals including (C) DA1 and (E) DL3 in the adult antennal lobe of control animals. α-sNPF immunoreactivity appears comparable to controls in the (D) DA1 and (F) DL3 of *Eph* null mutants. α-Brp (red) labels the neuropil. (G–H) Targeted expression of Eph in the olfactory sensory neurons does not change their overall pattern and OSNs appear comparable to (G) controls. (I–J) Uniglomerular projection neurons appear normal in *Eph* null animals. (I) Innervation pattern of projection neurons innervating glomeruli VA1d and DA1 in *Mz19*mCD8::GFP animals is unchanged in (J) in *Eph* null mutants (*Mz19*mCD8::GFP; *Eph^X652^*).

Eph-ephrin interactions can lead to diverse outcomes in terms of attraction, repulsion and cell adhesion in a context-dependent manner. High affinity Eph/ephrin signaling is known to initiate contact-dependent repulsion while low level signaling can lead to attraction and directed neuronal branch extension [42], [43], [44], [45]. We further investigated how Eph/ephrin signaling levels could control the final arborization pattern of the CSDn. To achieve a complete loss of Eph-ephrin signaling we utilized an allele *Eph^X652^* in where Eph expression is completely abolished [38]; Figure S4. Since Eph is expressed in the OSNs, we first tested the role of Eph during the development of OSNs and projection neurons (PNs), the primary synaptic partners of the OSNs. Terminals of OSNs (Figure 4A–4F) and uniglomerular PNs (Figure 4I–4J) develop normally in *Eph* null animals suggesting that Eph is not necessary for development of these components of the AL circuit, which have uniglomerular projections. Next, we asked if misexpression of Eph in the majority of the OSNs during a time window when Eph is expressed in very few glomeruli would affect OSN patterning in the AL. To this end, we used *Or83b*Gal4 [46], which drives Gal4 expression in ∼80% of the OSNs starting from mid-metamorphosis. Misexpression of Eph using *Or83b*Gal4 did not affect OSN patterning in the AL (Figure 4G, 4H). Overall, these observations allow us to argue that Eph signaling does not play any obvious role in OSN/uniglomerular PN patterning within the AL.

**Figure 5:**
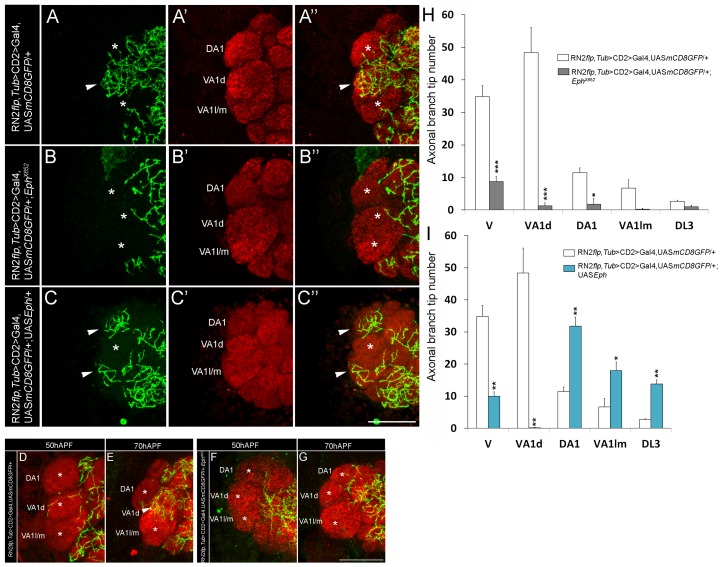
Perturbation in Levels of Eph signaling leads to defective glomeruli-specific positioning of the terminal of the CSDn. (A-A″, H) Innervation pattern of the axonal terminals of the CSDn (green) in glomeruli VA1l/m, VA1d and DA1 (anti-Brp in red) in control adults is shown (n≥6). (B-B″, H) In *Eph* null animals, axonal terminals of the CSDn show overall reduction in their AL innervation. This defect is pronounced in glomeruli which normally receive more innervations from the CSDn (VA1d (n = 4, p<0.001), VA1l/m (n = 4, p = 0.127), DA1 (n = 4, p = 0.025), DL3 (n = 4, p = 0.745) and V (n = 4, p<0.001). (C-C″, I) Targeted expression of Eph in the CSDn results in exquisite reversal of the terminal arborization pattern in these glomeruli compared to controls; terminals preferentially target VA1l/m (n = 5, p = 0.002), DA1 (n = 5, p<0.001), DL3 (n = 5, p = 0.003) and avoid glomerulus VA1d (n = 5, p<0.001). (H–I) Quantification of total axonal branch tip number is plotted in a histogram. Asterisks indicate glomeruli with fewer innervations and arrowhead indicates glomerulus with more innervations from the CSDn. A one-way repeated measure ANOVA test was performed to assess significant difference between the genotypes (F = 27.341, P<0.001). All pairwise multiple comparisions were performed using Fisher LSD method. *, p<0.05; **, p<0.01; ***, p<0.0001; n.s. (not significant), p>0.05. Scale bar = 20 µm. (D–G) Glomeruli-specific innervation of axonal terminals is achieved by directed growth of axonal terminals of the CSDn. Terminal arbors of the CSDn in (D–E) control (RN2*flp*, *tub*>CD2>Gal4, UAS*mCD8GFP*/+) and (F–G) *Eph* mutant animals (RN2*flp*, *tub*>CD2>Gal4, UAS*mCD8GFP*/+; *Eph^X652^*). Developmental profile of the axonal terminals of control CSDn at (D) 50 hAPF and (E) 70 hAPF is shown. (D) At 50 hAPF, very few axonal terminals of the CSDn can be seen extending to region of the AL where VA1l/m, VA1d, DA1 and DL3 are located. (E) Adult-like pattern of glomeruli-specific innervation of axonal terminals is apparent at 70 hAPF where high innervation of VA1d and low innervation of VA1l/m and DA1 by the CSDn terminals is seen. (F) At 50 hAPF, axonal terminals of the CSDn in *Eph* null mutants can be seen near the region of AL where the above-mentioned four glomeruli are located but (G) fail to innervate these glomeruli even at 70 hAPF. Asterisks indicate glomeruli with fewer innervations and arrowhead indicates glomerulus with more innervations from the CSDn. Scale bar = 20 µm.

Surprisingly, terminal innervations of CSDn were reduced in animals homozygous for *Eph^X652^* to all the glomeruli examined (Figure 5B, 5H and Table S1). This was in marked contrast to the situation where Eph-ephrin signaling was not completely abolished but only reduced in the *Ephrin* hypomorphs (Figure 1B) or where Eph was knocked down specifically in the OSNs (Figure 3C). The CSDn innervation pattern was differentially affected in the latter cases and glomeruli with normally less innervations showed a substantial increase, leaving the densely innervated glomeruli unaffected. These differences in phenotypes indicate a requirement of Eph signaling at multiple stages of the CSDn development. Complete loss of Eph throughout development might influence overall branching and hence we observed reduced arborization of the CSDn in *Eph* null. On the other hand, OSN-derived Eph controls glomerular-specifc innervation of the CSDn during pupal stages. In any event, our observations suggest a key role for Eph/ephrin pathway in patterning axonal terminals of the CSDn. To further test this, we ectopically expressed Eph in the CSDn. Targeted ectopic expression of Eph in the CSDn resulted in striking reversal of axonal branch tip distribution in the glomeruli (Figure 5C, 5I and Table S1). Axonal terminals of Eph-expressing CSDn preferentially innervated glomeruli with high Eph and completely avoided VA1d glomerulus, which expresses low Eph (Figure 5C, 5I). This exquisite mistargeting further strengthens the suggestion that levels of Eph/ephrin signaling control glomerular-specific innervation of this serotonergic neuron. One possibility is that preferential targeting to high Eph-expressing glomeruli could be due to attractive homotypic interactions between Eph expressing neurons. Eph-mediated homotypic interactions have been shown to promote cell adhesion between Eph-expressing cells during rhombomere-boundary formation in zebrafish [47]. Another possibility, not excluding the first, is that Eph-ephrin interaction within CSDn could result in ‘*cis* inhibition’ [28], [48] of the signaling pathway due to simultaneous presence of Eph and ephrin in the same cell, which in turn could reduce repulsive interaction and increase the attractive one.

We next examined if the developmental timing of the CSDn arborization is consistent with OSN derived Eph playing a role in the process. Glomerular-specific innervation of the CSDn involves directed growth of terminals to the target glomeruli. At 50 h after puparium formation (APF), very few arbors of CSDn were seen in the regions of the antennal lobe where VA1l/m, VA1d, DA1 and DL3 glomeruli were developing (Figure 5D). An adult-like pattern was seen by 70 h APF without an intermediate stage where excess arbors were seen (Figure 5E). Terminals of the CSDn failed to innervate these glomeruli in *Eph* null animals (Figure 5F–5G). The time course of the development of glomerular-specific arborization of the CSDn coincided with the expression profile of Eph, described above and is consistent with a role for Eph/ephrin pathway as regulators of this process. These observations demonstrate that the final arborization of the CSDn is not an outcome of excess growth in every glomerulus, followed by pruning but is an outcome of the repulsive signaling operating in high-Eph expressing glomeruli, which restrict the growth of CSDn terminals during development.

### CSDn regulates olfactory sensitivity of behaving animals in an odorant-dependent manner

**Figure 6:**
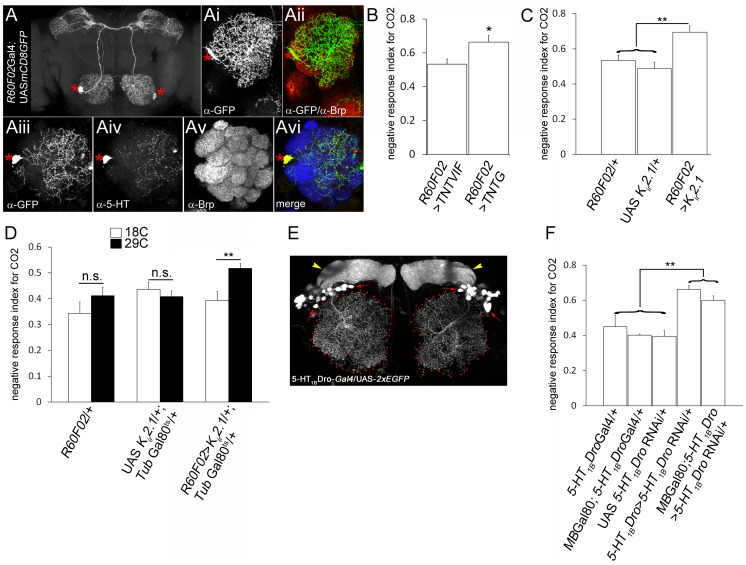
CSDn modulates odour-guided behaviour. The pair of CSDn is specifically labeled by *R60FO2*Gal4. Targeted expression of GFP using *R60FO2*Gal4 (*R60F02*Gal4/+; UAS *mCD8GFP*/+) shows a pair of neurons with anatomy characteristic of the CSDn and (Ai–Aii) innervations to the antennal lobe (Brp in red; GFP in green). (Aiii–vi) The neurons labeled by *R60F02* co-express 5-HT (red) indicating it is indeed the CSDn (green; Brp in blue). Asterisks indicate cell body of the CSDn. (B–F) The CSDn modulates olfactory response of adult *Drosophila* towards CO_2_. (B) Suppression of evoked synaptic transmission by targeted expression of tetanus toxin light chain (TNTG) in the CSDn (*R60F02*Gal4/+; UAS *TNTG*/+, n = 10, p = 0.017) leads to an increase in CO_2_ avoidance index compared to control animals (*R60F02*Gal4/+; UAS *TNTVIF*/+, n = 12). (C) Similar increase in CO_2_ sensitivity is observed upon suppression of CSDn excitability by targeted K_ir_2.1 expression (*R60F02*Gal4/+; UAS *K_ir_2.1*/+, n = 11, p<0.01 compared to controls) in the CSDn. (D) CSDn function is required in the adults for modulating olfactory behaviour. Adult-specific expression of K_ir_2.1 in the CSDn is achieved by rearing animals (*R60F02*Gal4/+; UAS *K_ir_2.1*/+; Tub-*Gal80^ts^*/+) at 18°C throughout development (white bars in D) and then shifting to 29°C after eclosion (black bars in D). Adult-specific suppression of CSDn excitability results in increased CO_2_ avoidance (n = 17; p = 0.006). (E) In a reporter line for serotonin receptor 5-HT_1B_Dro (*5-HT_1B_Dro*-Gal4/+; UAS-*2xEGFP*/+), a group of local interneurons are labeled (red arrows) along with mushroom bodies (yellow arrowheads). (F) RNAi-mediated knock down of 5-HT_1B_Dro in the 5-HT_1B_Dro expression domain (*5-HT_1B_Dro*-Gal4/+; UAS-*5-HT_1B_DroRNAi*/+, n = 11) results in increased CO_2_ sensitivity (p<0.05 compared to all control genotypes, n>7). 5-HT_1B_Dro expression outside the mushroom bodies, likely in the AL, may be necessary for CO_2_ sensitivity as blocking *5-HT_1B_DroRNAi* expression in mushroom body neurons (MB-Gal80/+; *5-HT_1B_Dro*-Gal4/+; UAS-*5-HT_1B_DroRNAi*/+, n = 14) does not ameliorate increased CO_2_ sensitivity (p = 0.12 compared to *5-HT_1B_Dro*-Gal4/+; UAS-*5-HT_1B_DroRNAi*/+, n = 11) and animals exhibit increased CO_2_ avoidance (p<0.01 compared to all control genotypes, n>7). Significance was assessed by *Mann-Whitney* test. *, p<0.05; **, p<0.01; ***, p<0.0001; n.s. (not significant), p>0.05.

We next examined if the extent of glomerular-specific arborizations of the CSDn has functional implications in behaving animals. To address this, an understanding of the role of the CSDn in odor-guided behaviours in *Drosophila* is first required. The CSDn is the only identified source of serotonin in the *Drosophila* AL [18], [49] suggesting an important role for this neuron in modulating olfactory perception. Although functional imaging studies have demonstrated that serotonin can change response properties of neurons in the AL [16], a direct demonstration of behavioural requirement of this neuron is lacking. We used the *R60F02*Gal4 strain [50] which consistently labels the CSDn bilaterally in the adult brain (Figure 6A), providing an advantage over the *cis*-FRT/FLP method, for behavioural analysis. *R60F02*Gal4's restricted expression in the central brain, with prominent expression in the CSDn and only a few arborizations in the suboesophageal ganglion provides an excellent reagent for behavioural experiments (Figure 6A). We validated that *R60F02*Gal4 indeed labels the CSDn in two ways. Firstly, the anatomy of its projections (Figure 6A, 6Ai and 6Aii) was similar to the described characteristic anatomy of the CSDn [18]. Furthermore by examining serotonin immunoreactivity in a genetic background where *R60F02*Gal4 expresses GFP, it was found that the only serotonin positive neuron in the AL co-localized with the GFP (Figure 6Aiii–vi) confirming that the Gal4 indeed specifically labels the CSDn.

For behavioural analysis, we selected two odorants; CO_2_ (perceived by the low Eph-expressing V glomerulus) and cVA (perceived by the high Eph-expressing DA1 and DL3 glomeruli) as innervations of the CSDn in the cognate glomeruli have been characterized by us. The behavioural response of wild-type adult *Drosophila* towards these odorants and the underlying neural circuitry is understood in good detail [34], [36]. CO_2_ is a repulsive stress pheromone in flies and is sensed by the V glomerulus [36]. Blocking evoked neurotransmitter release from the CSDn by targeted expression of tetanus neurotoxin light chain [TNTG; 51] rendered animals behaviourally more sensitive towards CO_2_ and these animals exhibited increased repulsion to CO_2_ compared to controls (Figure 6B, p = 0.017). Further, suppressing excitability of the CSDn by ectopic expression of an inward rectifying human K^+^ channel, K_ir_2.1 [52] in the neuron resulted in an increased CO_2_ avoidance behaviour (Figure 6C, p<0.01). Perturbation of neuronal activity during development has known consequences on the dendritic pattern of the CSDn [18], [21] and could be argued that this affects the behaviour. In order to circumvent the behavioural effects deriving from a developmental requirement of neural activity we manipulated the CSDn activity only during adulthood by using the temperature-sensitive Gal80 repressor of Gal4 (Gal80^ts^) [53]. Adult-specific suppression of the CSDn excitability by overexpression of the K_ir_2.1 in adult flies lead to increased CO_2_ sensitivity (Figure 6D) suggesting that the CSDn function in modulating olfactory behaviour is required during adulthood. In order to further validate the view that behavioural defects are indeed through serotonin signaling, we analyzed the expression pattern and function of serotonin receptors in the AL and then manipulated them. A Gal4 reporter line for serotonin receptor 5-HT_1B_Dro [9] labels a small set of local interneurons in the adult AL (Figure 6E) suggesting that these neurons could be possible downstream target of serotonin released by the CSDn. RNAi-mediated knock down of 5-HT_1B_Dro [9] in 5-HT_1B_Dro expression domain lead to an increase in CO_2_ avoidance behaviour (Figure 6F). However, 5-HT_1B_Dro is also expressed in the mushroom body neurons [9], which are a crucial component of the olfactory circuit underlying olfactory learning and memory [54], [55]. In order to define better, the domain of 5HT_1B_Dro expression relevant in mediating CO_2_ avoidance behaviour, 5HT_1B_Dro levels were ‘knocked-down’ using an RNAi construct [9] driven by the *5-HT_1B_Dro*-Gal4 driver in a context where Gal80 repressor of Gal4 is expressed under a mushroom-body promoter [56]. These animals will have normal 5HT_1B_Dro in the mushroom body neurons, due to Gal80 repressing GAL4 expression in this tissue, but lowered expression in the olfactory local interneurons due to RNAi. Behavioural experiments show that these animals exhibit an increased CO_2_ avoidance behaviour. Taken together, these observations suggest that the CSDn releases serotonin as a neuromodulatory transmitter and serotonergic receptor-expressing local interneurons play an important role in CO_2_ sensitivity.

**Figure 7:**
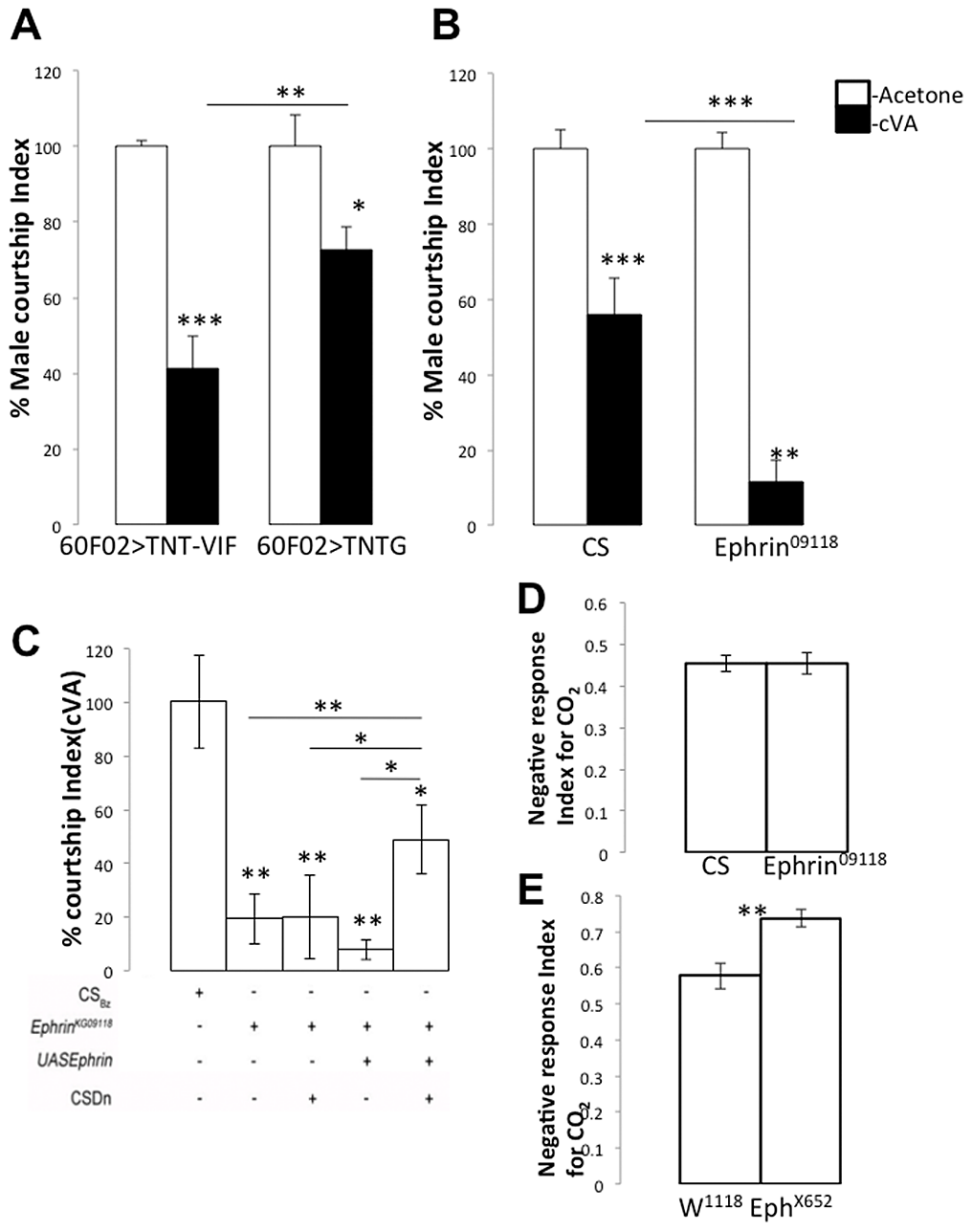
Glomerular-specific innervation pattern of the CSDn is relevant for odour-specific modulation of odour-guided behavior. (A) Targeted expression of tetanus toxin light chain in the CSDn (*R60F02*Gal4/+; UAS *TNTG/*+, n = 14, p = 0.028) leads to an increase in relative male courtship index towards cVA-treated virgin females compared to controls (*R60F02*Gal4/+; UAS *TNTVIF/*+, n = 16) which implies decreased sensitivity towards cVA in test animals. Now to test the effect of increasing arbors of the CSDn in cVA sensitive glomeruli we tested courtship index of *Ephrin^KG09118^*. (B) *Ephrin^KG09118^* males are more sensitive to cVA as exhibited by highly reduced courtship towards cVA-treated females (n = 25, p<0.001) compared to control males (n = 17). (C) Targeted Ephrin expression in the CSDn in an *Ephrin* mutant background (RN2*flp*, *tub*>CD2>Gal4, UAS*mCD8GFP*/UAS *Ephrin; Ephrin^KG09118^* in which both the CSD neurons were labeled; n = 26) results in partial rescue of the cVA sensitivity compared to the *Ephrin* mutant males (p = 0.004). . On the other hand, (D) CO_2_ sensitivity of *Ephrin^KG09118^* (n = 12; p = 0.98) is comparable to controls (n = 19). (E) *Eph^X652^* animals show increased avoidance (n = 8; p = 0.003) towards CO_2_ compared to controls (n = 10). As shown earlier, in *Eph^X652^* the CSDn innervations to V glomerulus are reduced while in *Ephrin* hypomorphs, these are comparable to controls. The courtship Index towards cVA treated females are normalized to the respective males Courtship Index towards Acetone (Mock) treated Virgin females. Significance was assessed by *Mann-Whitney* test. *, p<0.05; **, p<0.01; ***, p<0.0001; n.s. (not significant), p>0.05.

Next, we tested the role of the CSDn in cVA-dependent courtship behaviour. cVA, a male pheromone, is transferred to females during mating and renders them less attractive to other males in subsequent encounters. Virgin males therefore, show reduced courtship towards cVA-treated females [35]. The males sense the presence of cVA through OSNs that target to DA1 and DL3 glomeruli [34], [35]. Blocking neurotransmitter release from the CSDn by targeted expression of tetanus neurotoxin light chain in the CSDn resulted in reduced behavioural sensitivity towards cVA and these males exhibited increased courtship towards cVA-treated females compared to controls (Figure 7A, p = 0.028). Taken together, these experiments demonstrate a role for the CSDn in modulating olfactory perception of behaving animals in an odor-dependent manner.

### Differential glomerular innervation pattern of the CSDn affects odor-dependent behaviour modulation

Having established a role for the CSDn function in odor-response modulation, we examined the basis for this modulation. Modulation could be achieved in a variety of ways, such as the differential expression of serotonin receptors in the AL or/and by the differential arborization (as observed in the present study, Figure 1A), which in turn may result in differential levels of local serotonin release by the CSDn. Suppressing the function of the CSDn causes reduced behavioural sensitivity towards cVA, indicating that serotonin release is important for enhanced sensitivity towards cVA (Figure 7A). The level of serotonin release in the cVA-specific glomeruli (DA1 and DL3) is likely to be more in cases where there is an increase in the innervations of the CSDn to these glomeruli. Innervations in these glomeruli increase heavily in *Ephrin* hypomorphs compared to control (Figure 1) predicting that *Ephrin* hypomorphs should be much more sensitive to cVA. This was indeed the case; *Ephrin* hypomorphs showed a remarkable behavioural sensitivity to cVA and thus showed highly reduced courtship towards cVA-treated females (Figure 7B, p<0.001). If increased behavioural sensitivity in *Ephrin* hypomorphs is indeed due to increased DA1/DL3 innervations by the CSDn then rescuing the CSDn branching pattern to control levels should show a rescue of the behavioural phenotype. Targeted expression of Ephrin in the CSDn in *Ephrin* hypomorphs leads to a partial rescue of the behavioural sensitivity of *Ephrin* hypomorphs towards cVA (Figure 7C, p = 0.004 compared to *Ephrin* hypomorphs). This suggests that the increased sensitivity to cVA in the *Ephrin* hypomorphs is indeed due to the increased innervations of the CSDn. However, the absence of a complete rescue of the behavioural phenotype suggests the possibility that the terminals of other interneurons are defective in the relevant glomeruli in *Ephrin* hypomorphs. As mentioned earlier, the widespread expression of Ephrin in the lobe indicates that other interneurons may also require the molecule. Nevertheless, a partial behavioural rescue by Ephrin expression in the CSDn in *Ephrin* hypomorphs suggests that Eph/ephrin signaling has a role in development of the pheromone modulatory circuit and regulates correct positioning of neuronal arbors in a manner relevant for behaviour. Normal courtship in *Ephrin* hypomrphs (male courtship index = 0.72±0.032; n = 33) is comparable to controls (male courtship index = 0.76±0.037; n = 36) suggesting that these animals don't display a defect in courtship behaviour. A similar analysis could not be performed for *Eph* null animals as these showed severely reduced normal courtship (data not shown). We next checked whether this is true for the other odor we have examined, CO_2_. The CSDn innervations in the V glomerulus of *Ephrin* hypomorphs are comparable to controls (Figure 1) and their response towards CO_2_ is also comparable to control animals (Figure 7D, p = 0.98). However, *Eph^X652^* null animals, which have reduced innervations of the CSDn in the V glomerulus show an increased repulsion to CO_2_ when compared to controls (Figure 7E, p = 0.003). This phenotype is comparable to what we observed upon silencing or blocking neurotransmitter release from the CSDn (Figure 6). Thus, the olfactory sensitivity towards CO_2_ changes only in the contexts where the CSDn branching has been affected in V glomerulus. Taken together, our data suggests that the serotonergic CSDn has a modulatory effect in olfactory behavioural sensitivity and glomerular positioning of its terminals during development is essential for its function in the adult.

## Discussion

Our study demonstrates the Eph-ephrin dependent control of terminal-arborization pattern of an identified serotonergic neuron and shows that this regulation is put in place by sensory neuron derived Eph. Further, we link this Eph-ephrin mediated regulation of arborization to neuromodulation dependent behaviour. Eph-ephrin signaling in neural development has been studied [57], [58] but not at the level of an identified neuron and not in a serotonergic neuron. We suggest that such signaling, which combines repulsive as well as attractive responses could play a broad role in sculpting the target-domain of many wide-field neurons. Consistent with this view, we observe arborization defects in multi-glomerular interneurons when Eph-ephrin signal is compromised but not in typical PNs. PNs however show arborization defects upon misexpression of ephrin, demonstrating that Eph-ephrin repulsive signals can operate ectopically. Local targeting of neuronal arbors is a prerequisite to partner-specific connectivity and is thought to be achieved by differential expression of cell-surface molecules on pre- and post-synaptic cells [reviewed in 59], [60]. Eph/ephrin signaling could be part of a code of cell-surface molecules, which are thought to regulate local targeting and partner-specific neuronal connectivity in the AL [61].

### Regulation of interneuron innervation pattern by olfactory sensory neurons in the *Drosophila* antennal lobe

Several studies on the OSN and PN targeting in the *Drosophila* antennal lobe have led to a view that PNs organize the coarse map of the antennal lobe and thus provide spatial information necessary for appropriate fine-targeting of other lobe neurons [26], [62]. Glomerular organization of the antennal lobe is complete by ∼48 hAPF and synaptogenesis starts between 48 and 72 hAPF [63]. Our work shows that this developmental time window is not only relevant for synaptogenesis in the antennal lobe but also for OSN-driven patterning of wide-field interneurons. A small set of OSNs start to express Eph at the onset of the synaptogenic time window and provide spatial information to growing axonal terminals of the CSDn. Eph expressed by OSNs may not influence gross targeting of PNs as PN targeting occurs much earlier in the AL. However, OSN-derived Eph may regulate patterning of axonal terminals of other interneurons, which elaborate their branches during late metamorphosis. It will be interesting to see if selective Eph expression in the OSNs during the phase of synaptogenesis requires olfactory co-receptor expression or neuronal activity.

That the CSDn is a modified larval neuron [18] and that the glomerular-specific terminal pattern is set-up during pupal development both raise the possibility that serotonin release from this neuron has a role in antennal lobe development and plasticity. This possibility emerges from the very elegant set of studies from the Beltz laboratory [64], [65], which demonstrate a role of serotonin through its receptors, in adult neurogenesis in decapod crustaceans. One possibility is that the CSDn acts in the *Drosophila* larva to influence neurogenesis in the adult, during larval or pupal life, by regulating the specific LN and PN stem-cell linages and their neuronal morphogenesis in the antennal lobe [22], [23], 66. Another possibility, not excluding the first, is that serotonin is relevant to experience dependent changes in the glomeruli, such as observed in *Drosophila* [67]. We see no obvious alteration in the size of the antennal lobe in contexts where the CSDn function is blocked (data not shown) and, a detailed developmental role for serotonin is outside the scope of the current study. Nevertheless, the CSDn's singular presence in the antennal lobe make studying the developmental role of serotonin an attractive direction and an area that will surely be embarked on soon.

### Differential serotonergic modulation in the olfactory system

In most brain regions closely studied, each neuromodulatory transmitter is usually released by more than one neuron and co-expression with other neurotransmitters is not uncommon [68]. The *Drosophila* antennal lobe is likely to be similar and a recent study using mass- spectrometry and genetic tools suggests presence of a large number of neuromodulators in the AL [69]. This makes the linking of the development of identified neurons with their role in behaviour difficult to tease apart. The CSD neuron is special in that it is the only serotonergic neuron that innervates the AL and does not appear to have a co-transmitter. The CSD neuron preparation is thus valuable in that it allows the examination of neuromodulation from development of its anatomy to the role of this anatomy in behaviour. While there may well be a matrix of neuromodulators which together function in the behavioural paradigms we have tested, our results on ablating the function of the CSDn suggest that this neuron is likely to be a key player. Serotonergic neurons usually have a diffuse neuromodulatory role in the CNS. In such contexts, serotonin is able to diffuse from the release site in order to act upon extra-synaptic receptors and serotonergic neurons often branch in a manner to have complete coverage of the neuropil [2], [14], [27], [70]. The context-dependent response to serotonin is mediated by multiple serotonin receptors, which initiate diverse intracellular signal transduction pathways and also differ in their expression pattern in the central nervous system [71], [72]. Our analysis at the resolution of an identified neuron suggests that contextual specificity is also regulated at the level of innervation pattern and connectivity of serotonergic neurons. Our data points to a general mechanism underlying the emergence of contextual specificity in neuromodulation: peripheral neurons developmentally regulate the extent of innervation by modulatory neurons, which in turn, regulate the extent of neuromodulation of specific sensory pathways and behavioural output in the adult.

Our preparation allows the study of neuromodulatory regulation at every scale—from developmental anatomy to behaviour—and does so at the level of a single, identified neuron. A key gap in our study, which we recognize and are addressing as a longer-term direction, is the absence of a neurophysiological response. Published evidence demonstrates the physiological consequences of ectopic serotonin on the antennal lobe. Dacks et al used a genetically encoded Calcium indicator G-CaMP [16] to examine the responses of PNs to ectopic administration of serotonin. They argue that for some odors, serotonin could function by increasing projection neuron sensitivity. Importantly, they show that for odors that activate a wide-range of glomeruli, serotonin enhances PN responses in only some of these glomeruli. The natural suggestion from our study is that this differential alteration of PN responses in a glomerular-specific manner could be, at least in part, due to the specific arborization pattern of the CSDn termini. The response to serotonin is complex and not restricted to PNs alone. Dacks et al also demonstrate that serotonin enhances the responses of inhibitory LNs too. They argue that the effect of serotonin observed on PNs could be an indirect consequence of GABA from inhibitory LNs pre-synaptically acting on OSNs whose modulated function alters PN response. Experiments to test this or related models of serotonin dependent neuromodulation are technically challenging, require substantial time: Such experiments will require, for example, the measurement of OSN, LN and PN physiology when 5-HT receptors are blocked or absent in LNs. For now however, our findings that the CSD neuron's arborization affects behaviour in a manner similar to that seen when its activity is blocked or when 5-HT_1B_Dro receptor levels are down-regulated combined with the studies from Dacks et al [16] strongly suggest that the developmental regulation of local serotonin activity on neurons of the antennal lobe is an important component in the fly's olfactory response.

Why might flies differentially modulate two different olfactory responses? While CO_2_ is a stress odor for the fruit fly, cVA detection provides information about its mate and thus, each eliciting very different kind of behavioural responses. The mechanism of olfactory processing of CO_2_ is distinct from that of most other odorants: olfactory perception of CO_2_ requires co-expression Gr21a and Gr63a which belong to the Gustatory Receptor (GR) family rather than the Olfactory Receptor (DOR) family [73], [74]. Further, CO_2_ and cVA-sensing OSNs exhibit differences in GABA_B_R expression and consequently employ heterogeneous GABA-mediated presynaptic gain control [75]. In a natural context in the wild, presence of multiple odorants is expected. Differential modulation of functionally distinct odor-processing pathways could be used to advantage for an animal in the wild allowing it to adapt and fine-tune innate behavioral responses according to its immediate environment. Our data points to an element in the complex set of parts which puts such a system in place during development. Another level of sophistication might be added to the olfactory circuit by differential expression of serotonin receptors.

## Materials and Methods

### *Drosophila* strains

RN2*flp*, *tub*>CD2>Gal4, UAS*mCD8::GFP* [18] and *w*; If/CyO, wg-Z; *tub84B*-FRT-stop-FRT-LexA::VP16, RN2*Flp* (this study; referred as RN2*flp*, *tub*>stop>LexA::VP16 in the manuscript) flies were used for labeling the CSDn. Gal4-*GH146* was a gift from RF Stocker [19]; Gal4-*LN1* and Gal4-LN2 were provided by Kei Ito [23]; Or83b-Gal4 was kindly provided by LB Vosshall [46]. *5-HT_1B_Dro*-Gal4 and UAS *5-HT_1B_Dro*-RNAi lines were kindly provided by Amita Sehgal [9]. *Eph^X652^*/CiD [38], UAS *Eph* and UAS *Ephrin* lines were kindly provided by JB Thomas [38], [76]. *lexAop-rCD2::GFP* was provided by Tzumin Lee [77]. *Pebbled*-Gal4 [78] was provided by Rachel Wilson. *R60F02-*Gal4 was a gift from Gerald Rubin and it was generated as described in [50]; the 892 bp enhancer fragment in R60F02 derives from the acj6 gene and is delineated by the PCR primers caccagtgtcctgccggcgggcgaaaaga and aggtgccgcaatggaagtccttttt. UAS *TNTG* and UAS *TNTVIF* were kindly provided by Sean Sweeney [51]; UAS *Kir2.1* was a gift from Richard Baines [52]. *amos^1^/Df(2L)M36F-S6* was provided by Andrew German [39]. *wg^lacZ^* was a gift from JK Roy [79]. MB-Gal80 [56] was a gift from Andre Fiala. *Antp*, *Ephrin^KG09118^* [38] and Or47b-CD2 were obtained from Bloomington *Drosophila* stock center, Indiana University, USA. *wg^1-16^* was obtained from the *Drosophila* genetic resource, Kyoto, Japan. UAS *EphRNAi* (v4771) was obtained from the Vienna *Drosophila* RNAi Center [80]. All flies were maintained under standard conditions at 25°C unless otherwise indicated. For pupal timing, white prepupae (0 h after puparium formation – 0 hAPF) were collected and placed on a moist filter paper in humid conditions. This stage lasts for about an hour, thus setting the accuracy of staging; the pupal stage lasts 100 hours under conditions in our laboratory.

### Construction of the tub>stop>LexA::VP16 transgene

The tub>stop>LexA::VP16 construct was created by replacing the Gal80 coding region of a pCasper-tub-Gal80 construct with >stop>LexA::VP16. Therefore the LexA::VP16 was amplified via Polymerase Chain Reaction using the pBluescript-LexA::VP16 vector (Lai and Lee, 2006) as a template with the following primers: forward primer-GGG CTA GAG CGG CCG CGG CTA GCG CTC GCG ATA AGC TT and reverse primer- CAA AGA TCC TCT AGA GCC CCC TAC CCA CCG TAC TC. The resulting NotI-NheI-LexA::VP16-Xba PCR fragment was ligated via NotI and XbaI in an open pCasper-tub-NotI-XbaI vector (all enzymes from NEB). A successful ligation was verified via sequencing. The minimal >stop> cassette was inserted via ligation using the NheI side in front of the LexA::VP16 coding region. The orientation of the cassette was verified via digestion and sequencing. DNA for injection was purified using a Qiagen Midi Kit and transgenic lines were generated by BestGene Inc., (Chino Hills, CA, USA).

### Immunohistochemistry and confocal imaging

Brains from 2–4 days old adults were dissected and stained as described in [81]. Primary antibodies used were mouse anti-Bruchpilot/mAbnc82 (1∶20; DSHB), rabbit anti-GFP (1∶10,000; Molecular Probes, Invitrogen, Delhi, India), rabbit anti-Dephrin [1∶1000; Kind gift from Andrea Brand, 82] and rabbit anti-Serotonin (1∶500; Sigma). Secondary antibodies used were Alexa 488, Alexa-568 and Alexa-647 coupled antibodies generated in goat (Molecular Probes; 1∶400). Samples were mounted between coverslips with a spacer in 70% glycerol. Optical sections of 1 µm step size were analyzed using Olympus Fluoview version 1.4a, ImageJ (http://rsb.info.nih.gov/ij/; Wayne Rasband, NIH, USA) and Adobe Photoshop 7.0 (Adobe Systems, San Jose, CA, USA).

### Ephrin-Fc immunostaining protocol and validation of ephrin-Fc

Ephrin-Fc fusion probe [kind gift from Alan Nighorn, 38] was used to visualize Eph receptor expression pattern in the developing antennal lobe. Protocol from Kaneko and Nighorn [83] was used for *Drosophila* pupal brains. Ephrin-Fc specifically recognizes *Drosophila* Eph [38] and Ephrin-Fc immunoreactivity is completely abolished in *Eph^X652^* mutant pupal brains (Figure S1).

### Quantification of axonal terminal branch tip number

Glomeruli were identified using the standard 3-D map of the *Drosophila* antennal lobe [84]. Quantification of axonal terminal branch tip number was carried out in ImageJ (http://rsb.info.nih.gov/ij/; Wayne Rasband, NIH, USA) using the particle analysis and cell counter plugin. Branch tips of the CSDn in the individual glomerulus were marked manually using the plugin. Data was analyzed and represented as histogram using Microsoft Excel and graphpad instat. Statistical significance was determined using *Mann-Whitney* test and one-way repeated measure ANOVA test using Sigmaplot software.

### Male courtship assay in response to cVA

The assay was performed as previously described [34]. We tested courtship response of individually reared 5–6 day old virgin males of desired genotypes when introduced to age matched virgin CSBz females (reared in vials with ∼10 females), which were applied 0.2 µl of cVA (Pherobank, Netherlands) (diluted in Acetone) or only Acetone (as control). Concentration of cVA used was 1∶100 unless mentioned in particular experiment. The courtship response was recorded by videotaping (Sony Handycam DCR DVD910E & Sony DSC H9) in a chamber (Diameter = 1.5 cm; Height = 5 mm) for 10 mins, from which the courtship index was calculated manually as described previously [34].

### Measuring olfactory response to CO2

CO_2_ response index of 4–5 day old flies were measured using an upright Y-Maze apparatus as described elsewhere [67]. CO_2_was drawn through one arm of the maze, and control air was drawn through the other arm. Flies starved overnight were allowed into the entry tube, and their preference for the arm with the CO_2_ (O) vs. the control arm with air (C) was quantified as a response index [RI; the difference in the number of flies in the CO_2_ and control arms as a fraction of the total flies RI = (O−C)/(O+C)]. Behavioural analysis was done in a double-blind manner.

## Supporting Information

Figure S1 Innervations of the CSDn to glomeruli DL3 and V in different genetic backgrounds. (A–H″) Innervation pattern of the axonal terminals of CSDn (green) in glomeruli DL3 and (I–P) V in the adult brain is shown (n>6). Genotypes are (A-A″, I-I″) RN2*flp*, *tub*>CD2>Gal4, UAS*mCD8GFP*/+, (B-B″, J-J″) RN2*flp*, *tub*>CD2>Gal4, UAS*mCD8GFP*/+; *Ephrin^KG09118^*, (C-C″, K-K″) UAS *Ephrin*/+; RN2*flp*, *tub*>CD2>Gal4, UAS*mCD8GFP*/+, (D-D″, L-L″) UAS *Ephrin*/+; RN2*flp*, *tub*>CD2>Gal4, UAS*mCD8GFP*/+; *Ephrin^KG09118^*, (E-E″, M-M″) RN2*flp*, *tub*>CD2>Gal4, UAS*mCD8GFP*/+; *Eph^X652^*, (F-F″, N-N″) UAS *Eph*/+; RN2*flp*, *tub*>CD2>Gal4, UAS*mCD8GFP*/+ (G-G″, O-O″) RN2*flp*, *tub*>STOP>LexA::VP16/+, lexAOp*CD2GFP*/+ and (H-H″, P-P″) RN2*flp*, *tub*>STOP>LexA::VP16/+, lexAOp*CD2GFP*/+; *Pebbled*-Gal4>UAS *EphRNAi*. Synaptic neuropil is labeled by anti-Brp (in red). All the images are oriented as indicated in P″. D, dorsal; M, medial.
(TIF)

Figure S2 RNA interferance and misexpression of Eph in sensory neurons demonstrate the specificity of Ephrin-Fc and UAS*Eph*. (A-A′) Eph is expressed in a glomerular-specific manner in the antennal lobe of the control animals (UAS*EphRNAi*/+) as revealed by the ephrin-Fc probe (red). Pupal brains (70 hAPF) are counterstained with Phalloidin (grey) and the antennal lobe is encircled with white dots. (B-B′) Targeted expression of *EphRNAi* in the sensory neurons (*Pebbled*-Gal4/+; UAS *EphRNAi*/+) leads to robust reduction in Eph expression in the antennal lobe. (C-C′) *Pebbled*-Gal4/+; UAS *Eph*/+ animals show Eph misexpression in complete AL demonstrating specificity of the reagents.
(TIF)

Figure S3 Targeted expression of Ephrin in the projection neurons (PNs) results in PN branching defects. (A-A″) In control animals (Gal4-*GH146*,mCD8::GFP/+), PN arbors (GFP; green in merge panel) innervate glomeruli DL3, DA1, VA1d and VA1l/m and distinct glomerular organization of PNs can be seen. Synaptic neuropil is labeled by anti-Brp (red in merge panel). (B-B″) Ephrin overexpression in PNs (Gal4-*GH146*,mCD8::GFP/UAS *Ephrin*) results in severe disruption of the overall pattern of PNs in the antennal lobe as compared with (A-A″) control. Very few PN arbors seem to innervate DA1, VA1l/m and DL3 glomeruli (red asterisks in A and B), which lead to reduced size and altered shape of these glomeruli compared to controls. VA1d sees to receive comparable PN arbors and its size is also comparable to control VA1d glomerulus. (C-C″) GH146+ve typical PNs do not innervate the V glomerulus and a few fine arbors of atypical PNs can be seen (red asterisk). (D-D″) Ephrin overexpression in PNs (Gal4-*GH146*,mCD8::GFP/UAS *Ephrin*) does not affect this pattern in V glomerulus (red asterisk) and the shape and size of the V glomerulus is also comparable to control.
(TIF)

Figure S4 Ephrin-Fc labeling in the antennal lobe is specific to Eph. Ephrin-Fc immunoreactivity in the (A-A′) AL of control animals (RN2*flp*, *tub*>CD2>Gal4, *UASmCD8GFP*/+) at 70 hAPF. (B-B′) Ephrin-Fc staining is abolished in *Eph* null mutants (RN2*flp*, *tub*>CD2>Gal4, UAS*mCD8GFP*/+; *Eph^X652^*) and no immunoreactivity is detected in the AL at 70 hAPF. (Asterisk in B indicates region that normally expresses Eph). White dots encircle the AL.
(TIF)

Table S1 Quantification of the axonal branch tip number of the CSDn in different glomeruli. The table shows quantification of the axonal branch tip number (mean±SEM (n)) of the CSDn in different glomeruli in different genetic backgrounds. Glomerulus mentioned in bold express high Eph during development. All the above mentioned genotypes in the table have RN2*flp*, *tub*>CD2>Gal4, *UASmCD8GFP* in the background.
(PDF)
