# Correction: Sensory Neuron-Derived Eph Regulates Glomerular Arbors and Modulatory Function of a Central Serotonergic Neuron

**DOI:** 10.1371/journal.pgen.1007083

**Published:** 2017-11-01

**Authors:** Ajeet Pratap Singh, Rudra Nayan Das, Gururaj Rao, Aman Aggarwal, Soeren Diegelmann, Jan Felix Evers, Hrishikesh Karandikar, Matthias Landgraf, Veronica Rodrigues, K. VijayRaghavan

One of the affiliations for the 4th author is not indicated. Aman Aggarwal is affiliated with National Centre for Biological Sciences, Tata Institute of Fundamental Research, GKVK Campus, Bangalore, India, and also with Manipal University, Manipal, India.
