# Supplementary figures and images for: Sensory Neuron-Derived Eph Regulates Glomerular Arbors and Modulatory Function of a Central Serotonergic Neuron
Source: PLoS Genet. 2013 Apr 18;9(4):e1003452. doi: 10.1371/journal.pgen.1003452 (PMC3630106; doi:10.1371/journal.pgen.1003452)

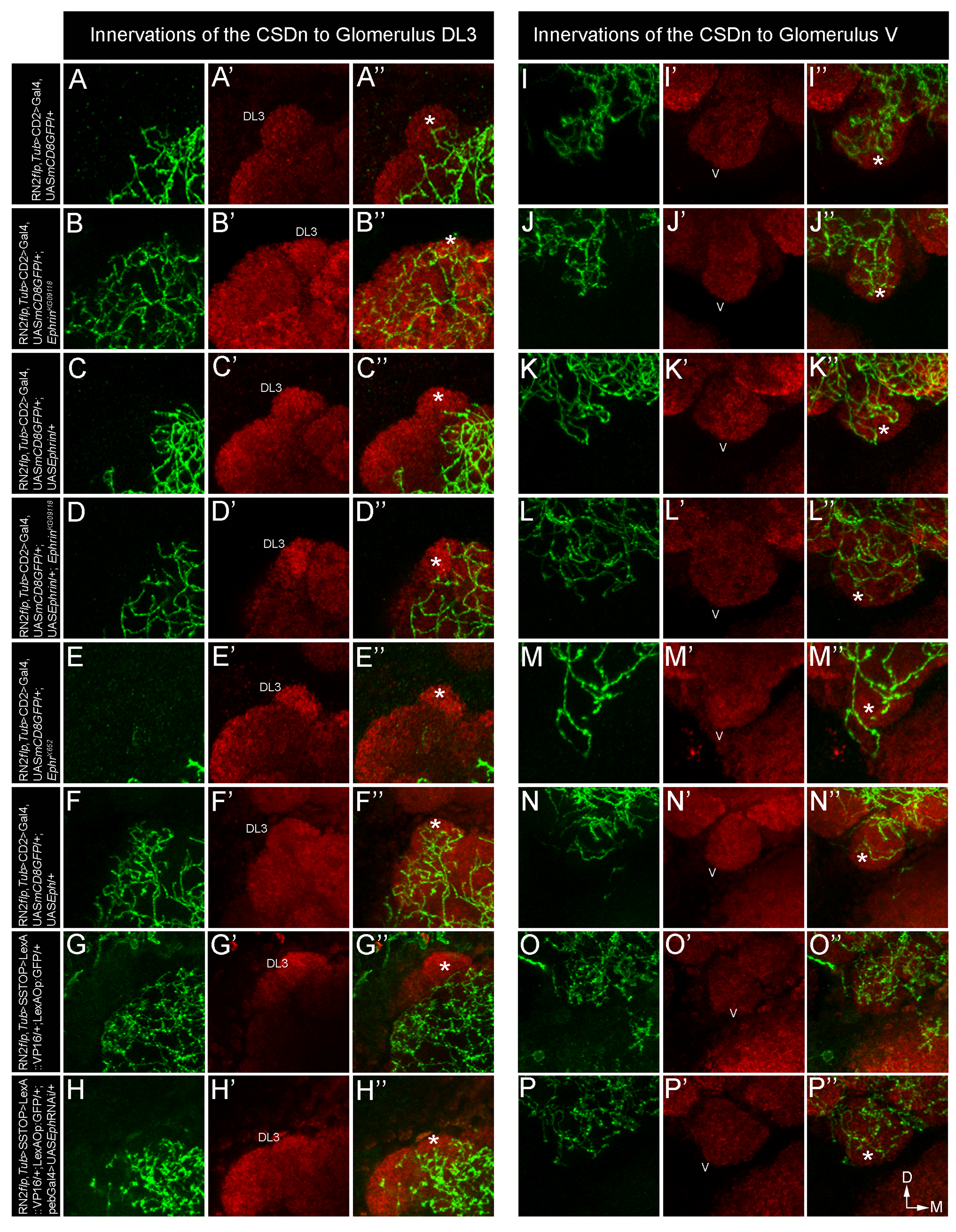

Supplement: Figure S1 — Innervations of the CSDn to glomeruli DL3 and V in different genetic backgrounds. (A–H″) Innervation pattern of the axonal terminals of CSDn (green) in glomeruli DL3 and (I–P) V in the adult brain is shown (n>6). Genotypes are (A-A″, I-I″) RN2flp, tub>CD2>Gal4, UASmCD8GFP/+, (B-B″, J-J″) RN2flp, tub>CD2>Gal4, UASmCD8GFP/+; EphrinKG09118, (C-C″, K-K″) UAS Ephrin/+; RN2flp, tub>CD2>Gal4, UASmCD8GFP/+, (D-D″, L-L″) UAS Ephrin/+; RN2flp, tub>CD2>Gal4, UASmCD8GFP/+; EphrinKG09118, (E-E″, M-M″) RN2flp, tub>CD2>Gal4, UASmCD8GFP/+; EphX652, (F-F″, N-N″) UAS Eph/+; RN2flp, tub>CD2>Gal4, UASmCD8GFP/+ (G-G″, O-O″) RN2flp, tub>STOP>LexA::VP16/+, lexAOpCD2GFP/+ and (H-H″, P-P″) RN2flp, tub>STOP>LexA::VP16/+, lexAOpCD2GFP/+; Pebbled-Gal4>UAS EphRNAi. Synaptic neuropil is labeled by anti-Brp (in red). All the images are oriented as indicated in P″. D, dorsal; M, medial. (TIF) [file pgen.1003452.s001.tif]

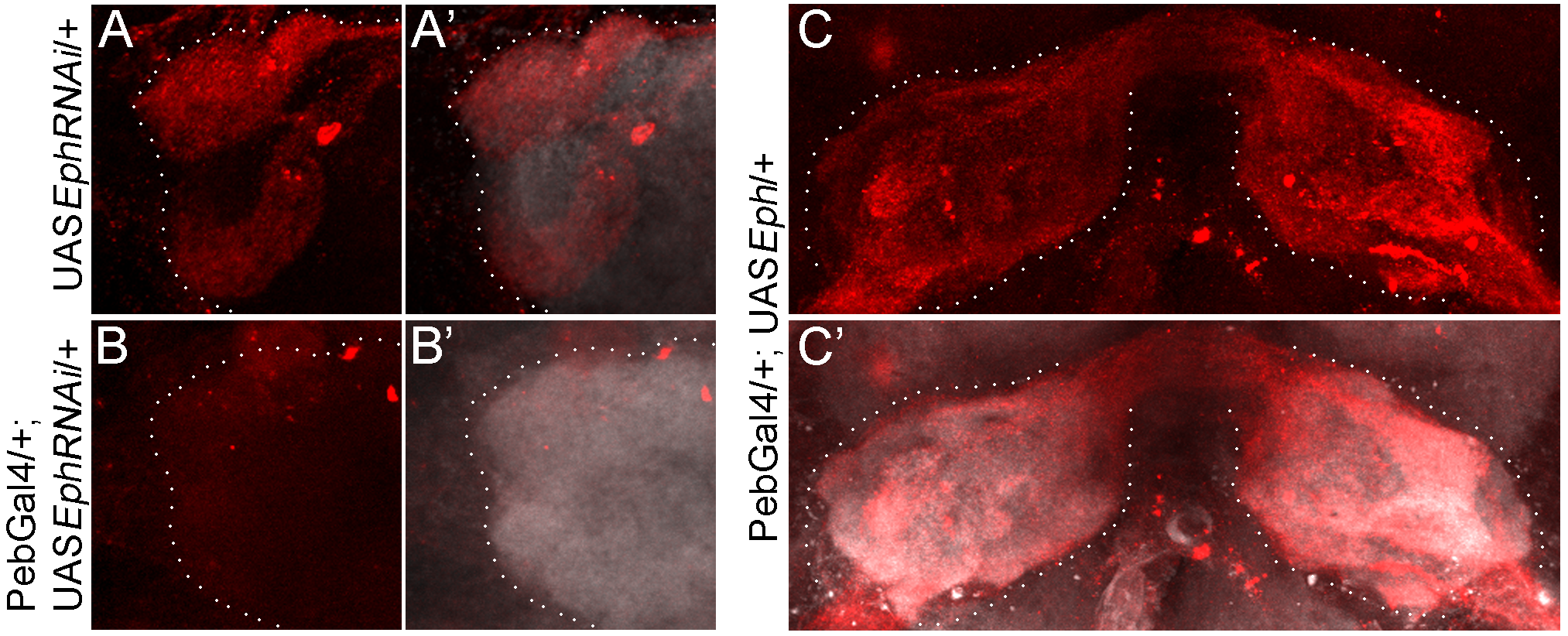

Supplement: Figure S2 — RNA interferance and misexpression of Eph in sensory neurons demonstrate the specificity of Ephrin-Fc and UASEph. (A-A′) Eph is expressed in a glomerular-specific manner in the antennal lobe of the control animals (UASEphRNAi/+) as revealed by the ephrin-Fc probe (red). Pupal brains (70 hAPF) are counterstained with Phalloidin (grey) and the antennal lobe is encircled with white dots. (B-B′) Targeted expression of EphRNAi in the sensory neurons (Pebbled-Gal4/+; UAS EphRNAi/+) leads to robust reduction in Eph expression in the antennal lobe. (C-C′) Pebbled-Gal4/+; UAS Eph/+ animals show Eph misexpression in complete AL demonstrating specificity of the reagents. (TIF) [file pgen.1003452.s002.tif]

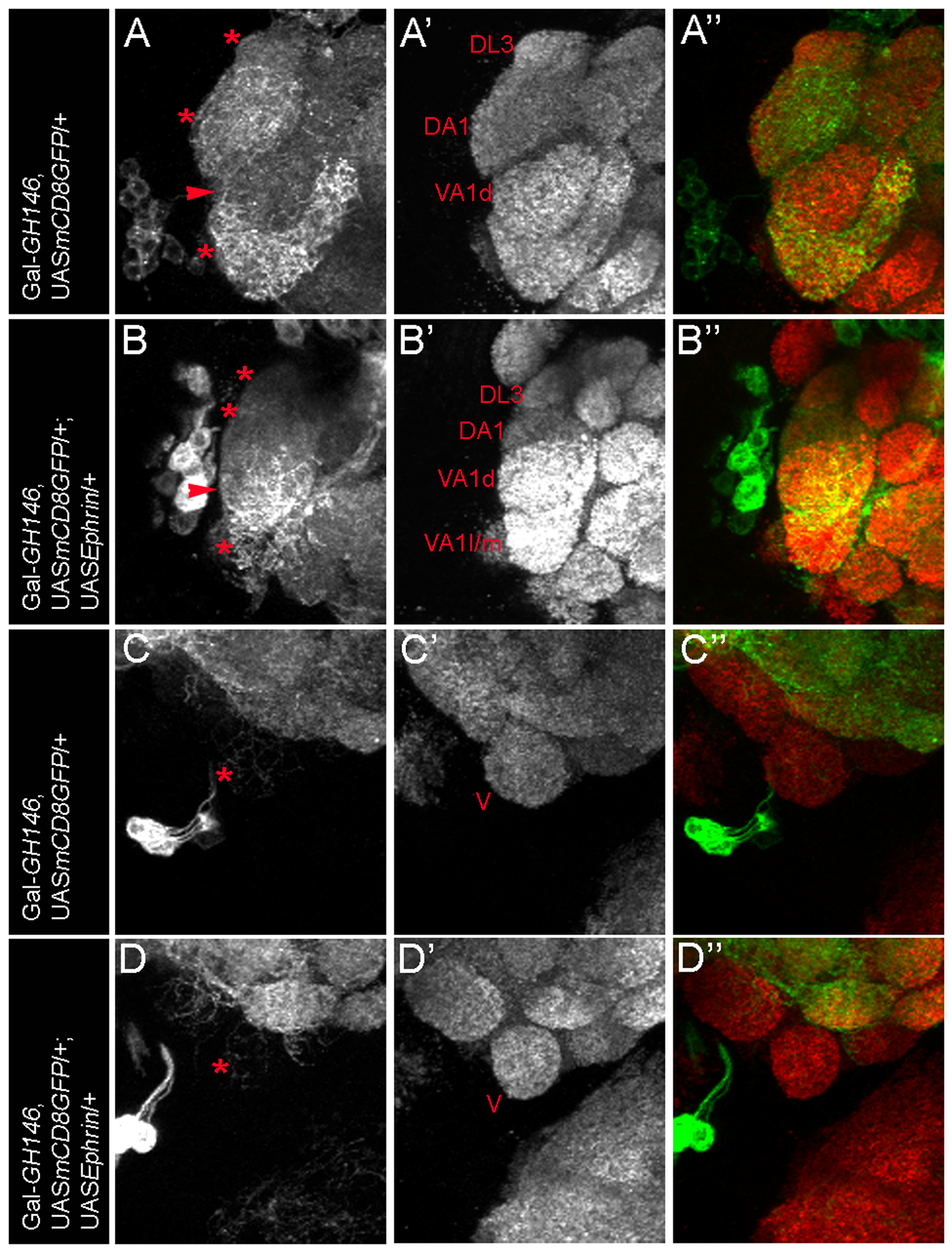

Supplement: Figure S3 — Targeted expression of Ephrin in the projection neurons (PNs) results in PN branching defects. (A-A″) In control animals (Gal4-GH146,mCD8::GFP/+), PN arbors (GFP; green in merge panel) innervate glomeruli DL3, DA1, VA1d and VA1l/m and distinct glomerular organization of PNs can be seen. Synaptic neuropil is labeled by anti-Brp (red in merge panel). (B-B″) Ephrin overexpression in PNs (Gal4-GH146,mCD8::GFP/UAS Ephrin) results in severe disruption of the overall pattern of PNs in the antennal lobe as compared with (A-A″) control. Very few PN arbors seem to innervate DA1, VA1l/m and DL3 glomeruli (red asterisks in A and B), which lead to reduced size and altered shape of these glomeruli compared to controls. VA1d sees to receive comparable PN arbors and its size is also comparable to control VA1d glomerulus. (C-C″) GH146+ve typical PNs do not innervate the V glomerulus and a few fine arbors of atypical PNs can be seen (red asterisk). (D-D″) Ephrin overexpression in PNs (Gal4-GH146,mCD8::GFP/UAS Ephrin) does not affect this pattern in V glomerulus (red asterisk) and the shape and size of the V glomerulus is also comparable to control. (TIF) [file pgen.1003452.s003.tif]

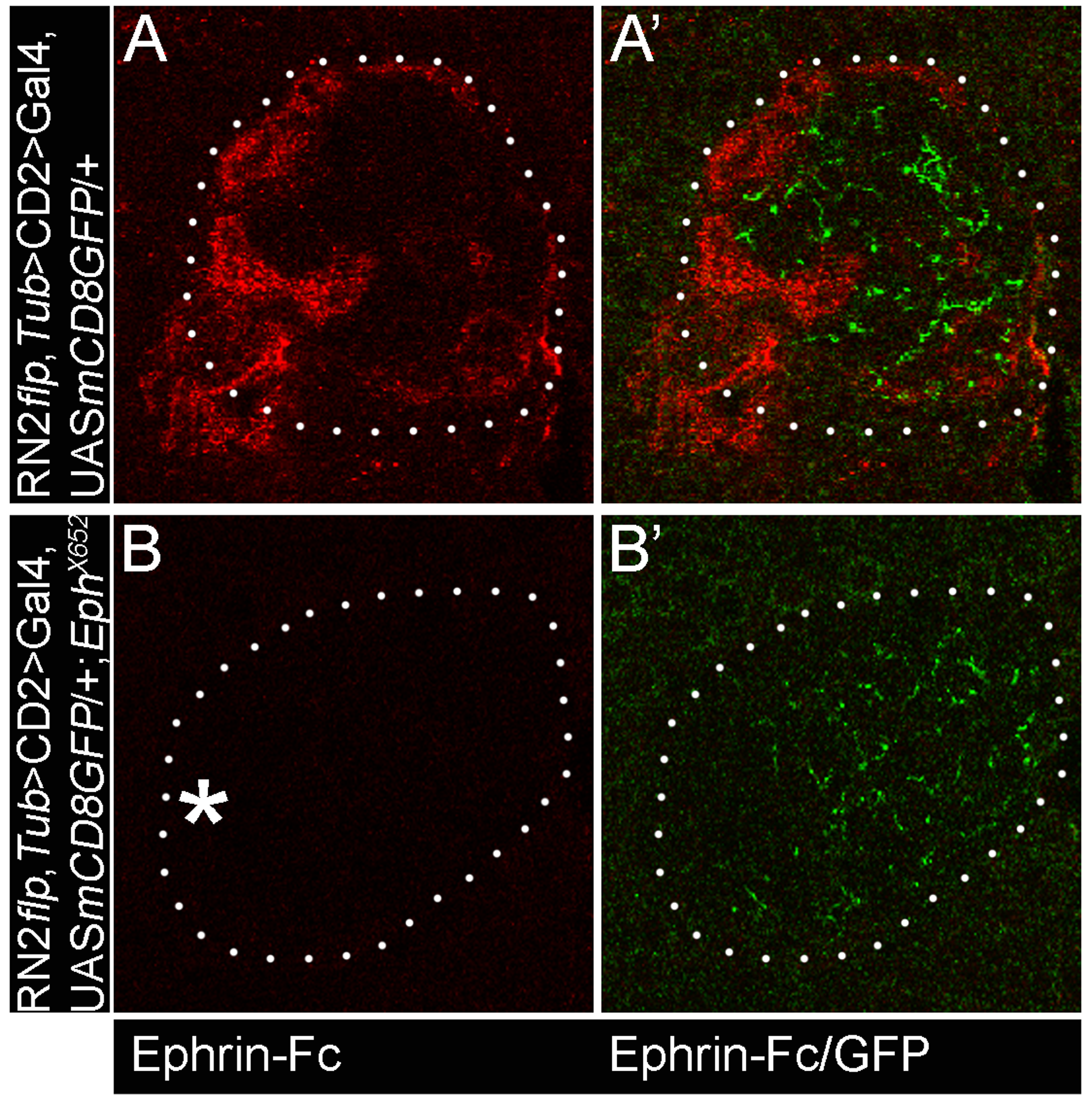

Supplement: Figure S4 — Ephrin-Fc labeling in the antennal lobe is specific to Eph. Ephrin-Fc immunoreactivity in the (A-A′) AL of control animals (RN2flp, tub>CD2>Gal4, UASmCD8GFP/+) at 70 hAPF. (B-B′) Ephrin-Fc staining is abolished in Eph null mutants (RN2flp, tub>CD2>Gal4, UASmCD8GFP/+; EphX652) and no immunoreactivity is detected in the AL at 70 hAPF. (Asterisk in B indicates region that normally expresses Eph). White dots encircle the AL. (TIF) [file pgen.1003452.s004.tif]
