# Supplementary material for: Sensory Neuron-Derived Eph Regulates Glomerular Arbors and Modulatory Function of a Central Serotonergic Neuron
Source: PLoS Genet. 2013 Apr 18;9(4):e1003452. doi: 10.1371/journal.pgen.1003452 (PMC3630106; doi:10.1371/journal.pgen.1003452)
Supplement: Table S1 — Quantification of the axonal branch tip number of the CSDn in different glomeruli. The table shows quantification of the axonal branch tip number (mean±SEM (n)) of the CSDn in different glomeruli in different genetic backgrounds. Glomerulus mentioned in bold express high Eph during development. All the above mentioned genotypes in the table have RN2flp, tub>CD2>Gal4, UASmCD8GFP in the background. (PDF) [file pgen.1003452.s005.pdf]

Table1. Quantification of the axonal branch tip number of CSDn in different glomeruli.

	wild type	*Eph*^{X652}	*Ephrin*^{KG09118}	UAS *Ephrin*	UAS *Ephrin*; *Ephrin*^{KG09118}	UAS *Eph*
VA1l/m*	*6.69±1.17(13)*	*0.25±0.25(4)*	*24.4±3.66(5)*	*2.5±2.48(8)*	*6.5±2.43(6)*	*19±5.96(5)*
VA1d	*48.33±7.8(6)*	*1.25±0.95(4)*	*49.2±4(5)*	*15.5±6.08(8)*	*17.0±5.62(6)*	*0.0±0.45(5)*
DA1*	*11.46±0.7(13)*	*1.75±1.75(4)*	*41.4±7.81(5)*	*4.0±3.25(8)*	*11.0±3.02(6)*	*32.0±6.18(5)*
DL3*	*2.69±0.35(13)*	*1.0±0.41(4)*	*9.89±0.72(9)*	*2.5±1.67(8)*	*2.5±1.05(6)*	*12.0±3.03(5)*
V	*34.83±3.41(6)*	*8.75±1.5(4)*	*28.0±4.42(4)*	*25.0±5.17(5)*	*21.0±4.8(5)*	*10.0±2.58(4)*

* Glomerulus in bold express high levels of Eph during development.

All the above mentioned genotypes in the table have RN2flp, tub>CD2>Gal4, UASmCD8::GFP in the background. Data is represented as mean±*SEM(n).*
